# 4-Chloro-2-(2-chloro­benzoyl)phenol

**DOI:** 10.1107/S1600536813025609

**Published:** 2013-09-21

**Authors:** A. Bushra Begum, S. Madan Kumar, B. C. Manjunath, Shaukath Ara Khanum, N. K. Lokanath

**Affiliations:** a Department of Chemistry, Yuvaraja’s College (Autonomous), University of Mysore, Mysore, 570 005, India; b Department of Studies in Physics, Manasagangotri, University of Mysore, Mysore, 570 006, India

## Abstract

In the title mol­ecule, C_13_H_8_Cl_2_O_2_, the dihedral angle between the benzene rings is 74.53 (9)°. An intra­molecular O—H⋯O hydrogen bond leading to a *S*(6) ring is observed. In the crystal, the mol­ecules are connected into a three-dimensional network by C—H⋯O and π–π [inter-centroid distance = 3.6254 (10) Å] inter­actions.

## Related literature

For the biological activity of benzo­phenone derivatives, see: Khanum *et al.* (2005 ▶, 2010 ▶). For a related structure, see: Devaiah *et al.* (2006 ▶).
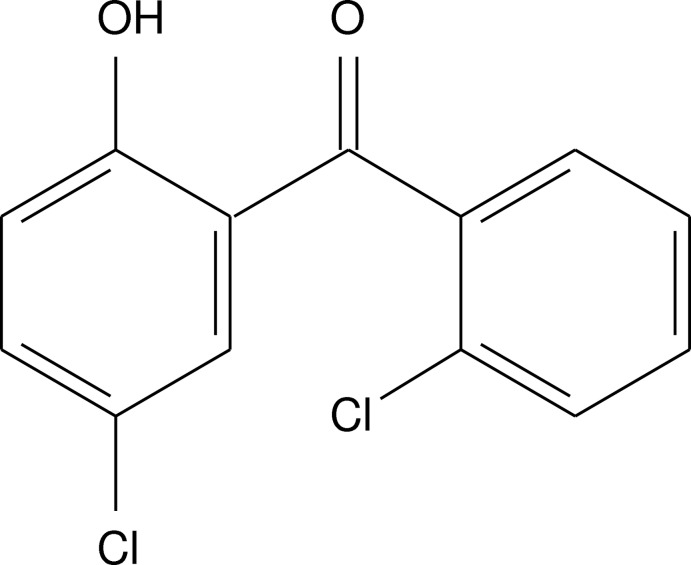


## Experimental

### 

#### Crystal data

C_13_H_8_Cl_2_O_2_
*M* *_r_* = 267.09
Orthorhombic, 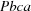

*a* = 16.0231 (4) Å
*b* = 7.4216 (2) Å
*c* = 19.6843 (5) Å
*V* = 2340.80 (10) Å^3^
*Z* = 8
Cu *K*α radiation
μ = 4.87 mm^−1^
*T* = 295 K
0.20 × 0.19 × 0.18 mm

#### Data collection

Bruker X8 Proteum diffractometer
Absorption correction: multi-scan (*SADABS*; Bruker, 2013 ▶) *T*_min_ = 0.442, *T*_max_ = 0.474
15868 measured reflections
1972 independent reflections
1712 reflections with *I* > 2σ(*I*)
*R*_int_ = 0.062

#### Refinement

*R*[*F* ^2^ > 2σ(*F* ^2^)] = 0.037
*wR*(*F* ^2^) = 0.105
*S* = 1.06
1972 reflections
154 parameters
H-atom parameters constrained
Δρ_max_ = 0.30 e Å^−3^
Δρ_min_ = −0.21 e Å^−3^

### 

Data collection: *APEX2* (Bruker, 2013 ▶); cell refinement: *SAINT* (Bruker, 2013 ▶); data reduction: *SAINT*; program(s) used to solve structure: *SHELXS97* (Sheldrick, 2008 ▶); program(s) used to refine structure: *SHELXL97* (Sheldrick, 2008 ▶); molecular graphics: *Mercury* (Macrae *et al.*, 2008 ▶); software used to prepare material for publication: *Mercury*.

## Supplementary Material

Crystal structure: contains datablock(s) global, I. DOI: 10.1107/S1600536813025609/tk5253sup1.cif

Structure factors: contains datablock(s) I. DOI: 10.1107/S1600536813025609/tk5253Isup2.hkl

Supplementary material file. DOI: 10.1107/S1600536813025609/tk5253Isup3.cml

Additional supplementary materials:  crystallographic information; 3D view; checkCIF report

## Figures and Tables

**Table 1:** Hydrogen-bond geometry (Å, °)

*D*—H⋯*A*	*D*—H	H⋯*A*	*D*⋯*A*	*D*—H⋯*A*
O16—H16⋯O9	0.82	1.88	2.598 (2)	146
C13—H13⋯O9^i^	0.93	2.50	3.413 (3)	168

Symmetry code: (i) 
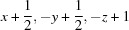
.

**Fig. 1.**
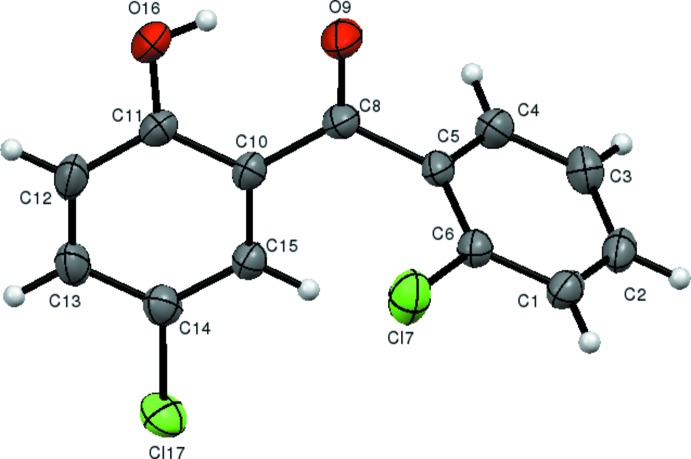
Molecular structure of the title compound along b-axis with 50% probability ellipsoids.

**Fig. 2.**
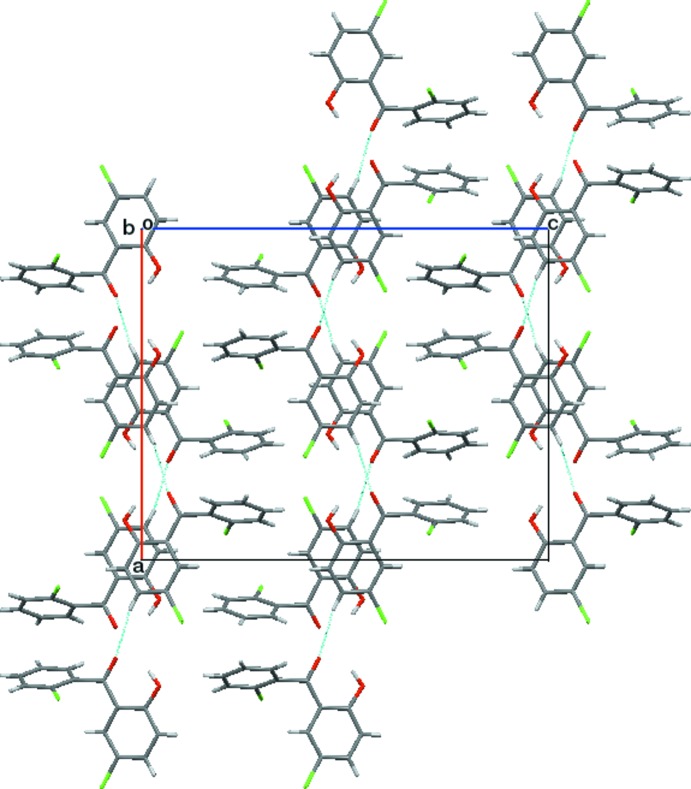
Packing diagram, viewed along the crystallographic b axis. Dotted lines represents C—H···O interactions.

## supplementary crystallographic information

### 1. Comment

The on-going research in synthesizing benzophenone derivatives in our laboratory resulted in the title molecule. These derivatives used in the preparation of anti-inflammatory (Khanum *et al.*, 2010) and anti-fungal (Khanum *et al.*, 2005) compounds.

In the title molecule (Fig. 1), the dihedral angle between chlorobenzene (C1–C6) and chlorohydroxybenzene (C10–C15) rings is 74.53 (9)°. The molecule features an intramolecular O—H..O hydrogen bond forming a *S*(6) ring (Table 1). The bond lengths and bond angles are similar to those in the 5-chloro-2-hydroxyphenyl-4-chlorophenyl-methanone structure (Devaiah *et al.*, 2006)

The molecules are connected by C13–H13···O9 hydrogen bonds forming chains along the *a* axis (Fig. 2 and Table 1). Additional C6—Cl7···π(Cg1), Table 1, and π(Cg2···π(Cg2) interactions, with inter-centroid distance 3.6254 (10) Å [*x*-1, -*y*, *z*-1], lead to a three-dimensional architecture, Fig. 2; where Cg1: C1–C6 and Cg2: C10–C15.

### 2. Experimental

A mixture of anhydrous aluminium chloride (1.74 g, 12.94 mmol) and include the name of the compound here (2.0 g, 8.62 mmol), was protected from moisture by a calcium chloride guard tube and heated over an oil bath at 80–90 °C for 45 min. At the end of this period the contents were cooled and decomposed by acidulated ice-cold water. The residual solid was crushed into a powder, dissolved in ether (40 ml) and extracted with 10% sodium hydroxide (3 x 30 ml). The basic aqueous solution was neutralized with 10% hydrochloric acid. The filtered solid was washed with distilled water (3 x 30 ml) and recrystallized from ethanol to afford yellow needles of the title compound. Yield 1.6 g (80%). M.Pt: 357–359 K. IR (Nujol): 1615 ν(C═O), 3525–3655 cm^-1^ν(OH). ^1^H NMR (CDCl_3_): δ 6.9–7.5 (m, 7H, Ar—H), 9.2 (bs, 1H, OH). EI–MS: m/*z* 267 (*M*+, 81), 266 (100), 154.5 (57), 111.5 (50). Anal. Calcd. for C_13_H_8_Cl_2_O_2_ (267): C, 58.46; H, 3.02; Cl, 26.55. Found: C, 58.54; H, 3.25; Cl, 26.32%.

### 3. Refinement

All the hydrogen atoms of the compound are fixed geometrically (C—H = 0.93–0.97 Å, O—H= 0.82 Å) and refined as riding with *U*_iso_(H) = 1.2 or 1.5 *U*_eq_(C, O).

### Crystal data

**Table:**

C_13_H_8_Cl_2_O_2_	*F*(000) = 1088
*M**_r_* = 267.09	*D*_x_ = 1.516 Mg m^−^^3^
Orthorhombic, *P**b**c**a*	Cu *K*α radiation, λ = 1.54178 Å
Hall symbol: -P 2ac 2ab	Cell parameters from 1972 reflections
*a* = 16.0231 (4) Å	θ = 4.5–64.9°
*b* = 7.4216 (2) Å	µ = 4.87 mm^−^^1^
*c* = 19.6843 (5) Å	*T* = 295 K
*V* = 2340.80 (10) Å^3^	Needle, yellow
*Z* = 8	0.20 × 0.19 × 0.18 mm

### Data collection

**Table:**

Bruker X8 Proteum diffractometer	1972 independent reflections
Radiation source: Bruker MicroStar microfocus rotating anode	1712 reflections with *I* > 2σ(*I*)
Helios multilayer optics monochromator	*R*_int_ = 0.062
Detector resolution: 10.7 pixels mm^-1^	θ_max_ = 64.9°, θ_min_ = 4.5°
\φ and \ω scans	*h* = −18→18
Absorption correction: multi-scan (*SADABS*; Bruker, 2013)	*k* = −8→4
*T*_min_ = 0.442, *T*_max_ = 0.474	*l* = −23→22
15868 measured reflections	

### Refinement

**Table:**

Refinement on *F*^2^	Primary atom site location: structure-invariant direct methods
Least-squares matrix: full	Secondary atom site location: difference Fourier map
*R*[*F*^2^ > 2σ(*F*^2^)] = 0.037	Hydrogen site location: inferred from neighbouring sites
*wR*(*F*^2^) = 0.105	H-atom parameters constrained
*S* = 1.06	*w* = 1/[σ^2^(*F*_o_^2^) + (0.0717*P*)^2^ + 0.4839*P*] where *P* = (*F*_o_^2^ + 2*F*_c_^2^)/3
1972 reflections	(Δ/σ)_max_ = 0.001
154 parameters	Δρ_max_ = 0.30 e Å^−^^3^
0 restraints	Δρ_min_ = −0.21 e Å^−^^3^

### Special details

**Table:**

Geometry. Bond distances, angles *etc*. have been calculated using the rounded fractional coordinates. All su's are estimated from the variances of the (full) variance-covariance matrix. The cell e.s.d.'s are taken into account in the estimation of distances, angles and torsion angles
Refinement. Refinement on *F*^2^ for ALL reflections except those flagged by the user for potential systematic errors. Weighted *R*-factors *wR* and all goodnesses of fit *S* are based on *F*^2^, conventional *R*-factors *R* are based on *F*, with *F* set to zero for negative *F*^2^. The observed criterion of *F*^2^ > σ(*F*^2^) is used only for calculating -*R*-factor-obs *etc*. and is not relevant to the choice of reflections for refinement. *R*-factors based on *F*^2^ are statistically about twice as large as those based on *F*, and *R*-factors based on ALL data will be even larger.

### Fractional atomic coordinates and isotropic or equivalent isotropic displacement parameters (Å^2^)

**Table:**

	*x*	*y*	*z*	*U*_iso_*/*U*_eq_	
Cl7	0.41862 (4)	0.46830 (6)	0.29403 (2)	0.0481 (2)	
Cl17	0.68664 (3)	0.11456 (7)	0.40130 (3)	0.0439 (2)	
O9	0.29828 (9)	0.2266 (2)	0.43513 (7)	0.0486 (5)	
O16	0.38062 (10)	0.3221 (2)	0.54298 (7)	0.0428 (5)	
C1	0.37955 (13)	0.1912 (2)	0.21207 (9)	0.0330 (5)	
C2	0.35120 (13)	0.0191 (2)	0.19888 (9)	0.0341 (5)	
C3	0.32654 (14)	−0.0927 (2)	0.25143 (10)	0.0376 (6)	
C4	0.33080 (13)	−0.0318 (2)	0.31807 (9)	0.0356 (6)	
C5	0.36117 (12)	0.1391 (2)	0.33254 (9)	0.0289 (5)	
C6	0.38485 (12)	0.2493 (2)	0.27869 (9)	0.0296 (5)	
C8	0.36471 (13)	0.1988 (2)	0.40532 (9)	0.0317 (5)	
C10	0.44516 (12)	0.2149 (2)	0.43988 (9)	0.0288 (5)	
C11	0.44873 (12)	0.2764 (2)	0.50758 (9)	0.0310 (5)	
C12	0.52597 (14)	0.2929 (2)	0.53977 (9)	0.0390 (6)	
C13	0.59771 (13)	0.2463 (3)	0.50739 (10)	0.0373 (5)	
C14	0.59454 (12)	0.1799 (2)	0.44109 (10)	0.0324 (5)	
C15	0.52003 (12)	0.1657 (2)	0.40762 (9)	0.0289 (5)	
H1	0.39490	0.26710	0.17660	0.0400*	
H2	0.34870	−0.02210	0.15430	0.0410*	
H3	0.30720	−0.20820	0.24220	0.0450*	
H4	0.31320	−0.10620	0.35330	0.0430*	
H12	0.52840	0.33640	0.58400	0.0470*	
H13	0.64880	0.25860	0.52940	0.0450*	
H15	0.51890	0.12320	0.36320	0.0350*	
H16	0.33910	0.30760	0.51930	0.0640*	

### Atomic displacement parameters (Å^2^)

**Table:**

	*U*^11^	*U*^22^	*U*^33^	*U*^12^	*U*^13^	*U*^23^
Cl7	0.0670 (4)	0.0413 (3)	0.0359 (3)	−0.0138 (2)	−0.0087 (2)	0.0009 (2)
Cl17	0.0269 (3)	0.0563 (3)	0.0484 (3)	0.0061 (2)	−0.0024 (2)	0.0039 (2)
O9	0.0283 (8)	0.0882 (10)	0.0294 (7)	0.0028 (7)	0.0032 (7)	−0.0086 (7)
O16	0.0401 (9)	0.0647 (8)	0.0237 (7)	−0.0061 (7)	0.0043 (6)	−0.0069 (6)
C1	0.0312 (10)	0.0440 (9)	0.0239 (9)	0.0027 (8)	−0.0001 (8)	0.0026 (7)
C2	0.0311 (10)	0.0465 (9)	0.0247 (9)	0.0068 (8)	−0.0053 (8)	−0.0055 (7)
C3	0.0368 (11)	0.0413 (9)	0.0347 (10)	−0.0027 (8)	−0.0084 (10)	−0.0037 (7)
C4	0.0332 (10)	0.0454 (10)	0.0281 (9)	−0.0050 (8)	−0.0019 (9)	0.0052 (7)
C5	0.0208 (9)	0.0437 (8)	0.0222 (8)	0.0011 (7)	−0.0026 (8)	0.0000 (6)
C6	0.0264 (9)	0.0381 (8)	0.0244 (8)	0.0004 (7)	−0.0020 (8)	−0.0006 (7)
C8	0.0262 (10)	0.0449 (9)	0.0241 (9)	0.0002 (7)	0.0020 (8)	0.0012 (7)
C10	0.0295 (10)	0.0349 (8)	0.0220 (8)	−0.0020 (7)	−0.0023 (8)	0.0030 (6)
C11	0.0331 (11)	0.0382 (8)	0.0216 (8)	−0.0044 (7)	0.0024 (8)	0.0022 (6)
C12	0.0465 (13)	0.0476 (9)	0.0228 (9)	−0.0109 (9)	−0.0073 (9)	0.0013 (7)
C13	0.0336 (10)	0.0475 (9)	0.0307 (9)	−0.0070 (8)	−0.0104 (9)	0.0074 (7)
C14	0.0291 (10)	0.0359 (8)	0.0322 (9)	−0.0006 (7)	−0.0036 (9)	0.0066 (7)
C15	0.0291 (10)	0.0353 (8)	0.0224 (8)	0.0005 (7)	−0.0009 (8)	0.0013 (6)

### Geometric parameters (Å, º)

**Table:**

Cl7—C6	1.7394 (16)	C10—C15	1.406 (3)
Cl17—C14	1.740 (2)	C10—C11	1.410 (2)
O9—C8	1.233 (2)	C11—C12	1.396 (3)
O16—C11	1.339 (2)	C12—C13	1.359 (3)
O16—H16	0.8200	C13—C14	1.396 (3)
C1—C6	1.383 (2)	C14—C15	1.368 (3)
C1—C2	1.380 (2)	C1—H1	0.9300
C2—C3	1.384 (3)	C2—H2	0.9300
C3—C4	1.389 (3)	C3—H3	0.9300
C4—C5	1.388 (2)	C4—H4	0.9300
C5—C6	1.392 (2)	C12—H12	0.9300
C5—C8	1.501 (2)	C13—H13	0.9300
C8—C10	1.462 (3)	C15—H15	0.9300
			
C11—O16—H16	109.00	C11—C12—C13	120.98 (17)
C2—C1—C6	119.15 (16)	C12—C13—C14	119.83 (19)
C1—C2—C3	120.55 (16)	Cl17—C14—C13	119.24 (15)
C2—C3—C4	119.79 (15)	C13—C14—C15	120.62 (18)
C3—C4—C5	120.55 (16)	Cl17—C14—C15	120.14 (15)
C4—C5—C8	118.62 (15)	C10—C15—C14	120.49 (17)
C6—C5—C8	122.91 (14)	C2—C1—H1	120.00
C4—C5—C6	118.45 (16)	C6—C1—H1	120.00
Cl7—C6—C5	120.12 (13)	C1—C2—H2	120.00
C1—C6—C5	121.49 (15)	C3—C2—H2	120.00
Cl7—C6—C1	118.36 (13)	C2—C3—H3	120.00
O9—C8—C10	121.73 (16)	C4—C3—H3	120.00
C5—C8—C10	120.10 (17)	C3—C4—H4	120.00
O9—C8—C5	118.11 (18)	C5—C4—H4	120.00
C8—C10—C11	120.13 (17)	C11—C12—H12	120.00
C8—C10—C15	121.39 (16)	C13—C12—H12	119.00
C11—C10—C15	118.46 (17)	C12—C13—H13	120.00
O16—C11—C10	122.76 (17)	C14—C13—H13	120.00
O16—C11—C12	117.67 (16)	C10—C15—H15	120.00
C10—C11—C12	119.57 (17)	C14—C15—H15	120.00
			
C6—C1—C2—C3	1.5 (3)	O9—C8—C10—C15	173.55 (16)
C2—C1—C6—Cl7	−178.65 (16)	C5—C8—C10—C11	178.00 (14)
C2—C1—C6—C5	−0.9 (3)	C5—C8—C10—C15	−3.7 (2)
C1—C2—C3—C4	−0.4 (3)	C8—C10—C11—O16	0.0 (2)
C2—C3—C4—C5	−1.3 (3)	C8—C10—C11—C12	−179.36 (14)
C3—C4—C5—C6	1.8 (3)	C15—C10—C11—O16	−178.36 (15)
C3—C4—C5—C8	−179.90 (19)	C15—C10—C11—C12	2.3 (2)
C4—C5—C6—Cl7	176.96 (15)	C8—C10—C15—C14	−179.27 (15)
C4—C5—C6—C1	−0.7 (3)	C11—C10—C15—C14	−0.9 (2)
C8—C5—C6—Cl7	−1.2 (3)	O16—C11—C12—C13	178.94 (17)
C8—C5—C6—C1	−178.93 (18)	C10—C11—C12—C13	−1.7 (2)
C4—C5—C8—O9	−69.0 (2)	C11—C12—C13—C14	−0.4 (3)
C4—C5—C8—C10	108.3 (2)	C12—C13—C14—Cl17	−178.09 (14)
C6—C5—C8—O9	109.2 (2)	C12—C13—C14—C15	1.8 (3)
C6—C5—C8—C10	−73.5 (2)	Cl17—C14—C15—C10	178.75 (12)
O9—C8—C10—C11	−4.8 (2)	C13—C14—C15—C10	−1.1 (2)

### Hydrogen-bond geometry (Å, º)

Cg1 is the centroid of the C1–C6 benzene ring.

**Table:**

*D*—H···*A*	*D*—H	H···*A*	*D*···*A*	*D*—H···*A*
O16—H16···O9	0.82	1.88	2.598 (2)	146
C13—H13···O9^i^	0.93	2.50	3.413 (3)	168
C6—Cl7···*Cg*1^ii^	1.74 (1)	3.89 (1)	4.901 (2)	116 (1)

Symmetry codes: (i) *x*+1/2, −*y*+1/2, −*z*+1; (ii) −*x*, *y*+1/2, −*z*+3/2.
